# Etiology and Management of Hemorrhagic Complications of Portal Hypertension in Children

**DOI:** 10.1155/2012/879163

**Published:** 2012-10-11

**Authors:** Alejandro Costaguta, Fernando Alvarez

**Affiliations:** ^1^Unidad de Hígado y Trasplante Hepático, Sanatorio de Niños, Alvear 863, Santa Fe, Rosario 2000, Argentina; ^2^Service de Gastroentérologie, Hépatologie et Nutrition, CHU Sainte-Justine, Université de Montréal, 3175 Côte Sainte-Catherine, Montréal, QC, Canada H3T 1C5

## Abstract

Portal hypertension in children represents a particular diagnostic and management challenge for several reasons: (1) treatment outcomes should be evaluated in relationship with a long-life expectancy, (2) pediatric patients with portal hypertension constitute an heterogeneous population, both in terms of individual characteristics and diversity of liver diseases; making comparison between treatment outcomes very difficult, (3) application of techniques and procedures developed in adult patients (v.gr. TIPS) face size limitations in small children, and (4) absence of data from well-controlled trials in children forces pediatric specialists to adapt results obtained from adult cohorts suffering from diseases such as HCV and alcoholic cirrhosis. Despite those limitations, substantial progress in the treatment of children with portal hypertension has been achieved in recent years, with better outcomes and survival. Two main factors influence our therapeutic decision: age of the patient and etiology of the liver disease. Therefore, diagnosis and treatment of complications of portal hypertension in children need to be described taking such factors into consideration. This paper summarizes current knowledge and expert opinion.

## 1. Presinusoidal Portal Hypertension

### 1.1. Portal Vein Obstruction

Portal obstruction is the single most common etiology of portal hypertension in children, representing roughly 50% of all cases in the majority of series. The causes of portal vein obstruction fall into one of following categories: perinatal events (umbilical catheterization, omphalitis, and dehydration), congenital malformations outside the portal vein (Abernethy malformation), thrombophilic states (deficiency of protein-C, S or antithrombin-III, etc.), tumors, abdominal infections, and a category where the etiology is unknown [1, 2].

Portal obstruction in children is usually detected early in the first decade, because of splenomegaly, gastrointestinal bleeding, or both [3]. Development of esophageal varices is almost universal, and the actuarial risk of bleeding reaches 76% at 24 years of age. Probability of bleeding is directly correlated with the size of varices as seen on endoscopy, from the absence of bleeding episode in children without varices or with grade I varices, to 85% prevalence of bleeding in patients with grade II or IIII varices, as reported by Lykavieris et al. [4]. Of note, this study showed that varices tended to increase in size over the years instead of disappearing, defying the classical concept of spontaneous improvement as children grow-up. 

**Figure 1:**
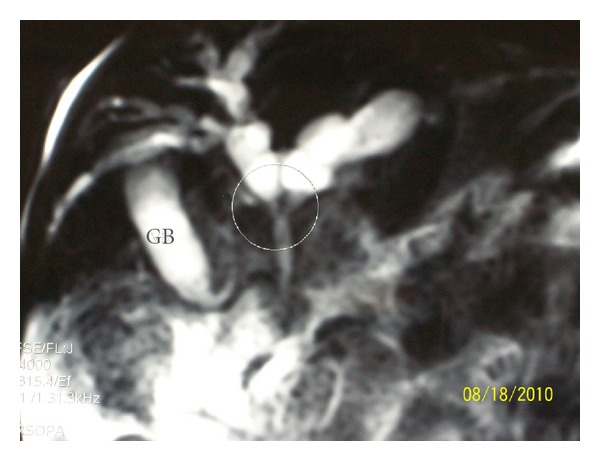
Portal cholangiopathy: this 18-year-old-boy presented with fever and jaundice. He has been treated with endoscopic sclerosis of esophageal varices from the age of six, because of portal hypertension secondary to extrahepatic portal vein obstruction. Cholangio-MRI shows dilated intrahepatic biliary tree, proximal to the level of stenosis (circle). GB: gallbladder.

Variceal bleeding is generally well tolerated, owing to normal function of the liver; however, the main concern in the management is to reduce the recurrence of episodes. Endoscopic therapy works by physical obliteration of esophageal varices and has shown excellent results, with a 90% rate of success in the long-term control of bleeding [5]. It usually represents the first approach due to its relative simplicity, low frequency of immediate complications, and widespread availability. The high rate of success has led to ample use of this technique; however, an increase of long-term complications is usually observed, as bleeding from ectopic varices, low-grade encephalopathy, hepatopulmonary syndromes, further development of hypersplenism, and cholestasis secondary to portal cholangiopathy. Particularly challenging is the management of cholestasis; this syndrome has been described in 6% of patients with portal vein obstruction, especially after long-term followup [6, 7], and it is the consequence of dilated peribiliary venous plexus (cavernoma) in the wall of biliary ducts (Figure 1). Affected patients exhibit high levels of GGT and Bilirubin, with dilated bile ducts (mainly intrahepatic) as seen on the abdominal ultrasound. Biopsy samples show different degrees of fibrosis and even biliary type of cirrhosis, with a pattern indistinguishably from primary sclerosing cholangitis in some cases [6].

Complete resolution can be achieved with surgical decompression of the portal system by means of a portosystemic or a meso-Rex shunts. In rare cases persistent biliary strictures remain present after shunt surgery. Probably ischemic in nature, they can be resolved by hepaticojejunostomy [7]. 

### 1.2. Congenital Hepatic Fibrosis

Congenital hepatic fibrosis (CHF) is part of a spectrum of fibropolycystic diseases, in which the pathological hallmark is the presence of *ductal plate malformation *[8]. It combines biliary dysplasia, perilobular fibrosis, and renal polycystic disease in different patterns, giving rise to a wide diversity of clinical manifestations observed throughout the years. Two different forms have been described in association with renal disease: autosomic recessive (ARPKD) and dominant (ADPKD) polycystic kidney diseases [9].

In ARPKD, clinical signs of renal disease can be observed during the first years, appear later, or remain subclinical. Findings of portal hypertension become evident, generally in the first years of life, usually in the form of variceal bleeding and hypersplenism. It has been estimated that 25% of affected individuals develop clinically significant portal hypertension, with a trend toward increased frequency with increasing age [10]. Interestingly, children with portal hypertension were younger than the mean age of the whole cohort, suggesting that a particular subset of patients is at risk of developing this complication, probably related to specific still unknown genetic or environmental factors.

ADPKD patients, in contrast with ARPKD, tend to present later in life with progressive renal disease and less liver involvement. However, because variceal bleeding can occur as early as age 4, screening relatives of the index case (most commonly an adult with multiple renal cysts) by regular ultrasounds have been recently advocated [11].

CHF has also been reported as part of other rare syndromes, such as nephronopthisis (with end-stage renal disease within 5 to 10 years), Jeune syndrome (lung and thoracic hypoplasia), Meckel-Gruber syndrome (encephalocele and polydactily), Ivemark syndrome (interstitial fibrosis leading to renal failure), chronic diarrhea related to enterocolitis cystic superficialis and intestinal lymphangiectasia, and others. In all cases, accompanying liver findings include ductal plate malformation, fibrosis, and biliary cysts in different combinations [12]. 

Patients with congenital hepatic fibrosis characteristically have well-preserved liver function; they behave as those with portal vein obstruction, with regard to the risk and tolerance to bleeding. Moreover, cavernomatous transformation of the portal vein and abnormal intrahepatic branching have been described in CHF patients, suggesting that anomalies in the development of portal veins are part of the spectrum of liver disease in this condition [13, 14].

Given the relatively benign liver disease, management recommendations for children with CHF-related portal hypertension are based on endoscopic eradication of varices. However, the frequent need for kidney transplantation in children with ARPKD leads to perform a surgical portosystemic shunt before the transplant surgery. Successful shunt facilitates abdominal surgery and avoids varices bleeding that could represent a risk for the transplanted organ. For the rare patients with repeated acute or chronic cholangitis, who develop cirrhosis, or for those with pulmonary complications, liver transplantation is a potential therapeutic option. Decision about when (and if) to combine it with kidney transplantation should be considered on a case-by-case evaluation [15].

## 2. Cholestatic Cirrhosis

### 2.1. Biliary Atresia

This disease affects 1 in 15000 to 1 in 20000 newborns and constitutes the main indication for liver transplantation in children. Current treatment strategy includes the Kasai portoenterostomy operation, followed by liver transplantation in cases of its failure or later complications from cirrhosis [16]. Children with biliary atresia tend to develop varices very early, with an estimated risk of bleeding of 15% before the age of two [17]. When associated with high bilirubin levels, it portends a poor prognosis, and constitutes an indication to proceed to transplantation as soon as possible, owing to the more than tenfold rise in the risk of death when conjugated bilirubin levels are over 10 mg% [18]. Even in anicteric patients, there is a considerable risk of bleeding, highlighting their tendency to suffer from severe portal hypertension, probably related to the intense fibrosis as is observed at the time of portoenterostomy, and the diffuse compromise of intrahepatic portal vein described in some [14]. Cholangitis, a frequent complication after portoenterostomy, can be responsible for thrombophlebitis of the portal system, accelerating the development of portal hypertension [19].

**Figure 2:**
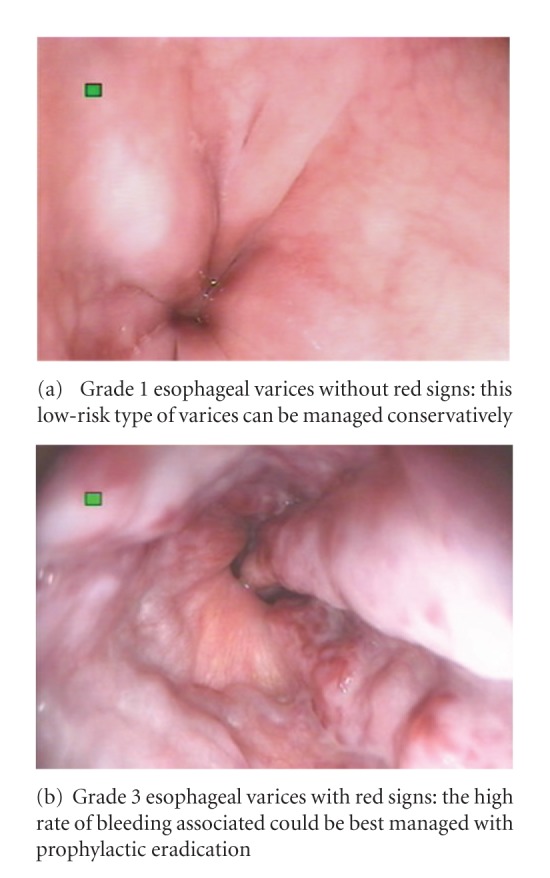
Different types of esophageal varices on endoscopic examination.

Bleeding can be predicted in patients with large varices, associated red signs, presence of gastric varices, and portal hypertensive gastropathy (Figure 2) [17]. Recent data supports the implementation of prophylactic sclerotherapy or banding to prevent the first hemorrhage. Endoscopy screening can be suggested to begin around 12 months of age. Sclerotherapy would be preferred over rubber band ligation owing to size constraints faced in little children [20]. 

### 2.2. Cystic Fibrosis

Approximately 5% of cystic fibrosis patients develop liver cirrhosis before adolescence [21].

Like other cholestatic type of cirrhosis, it is characterized by a high degree of portal hypertension, with preserved synthetic function for many years [22, 23]. As the management of lung disease continues to improve, liver disease is becoming a major determinant of the outcome, being the third most common cause of death [24]. It has been estimated that nearly 60% of cirrhotic patients experimented an episode of variceal bleeding before the second decade of life [23], contributing to the 10 to 20% of deaths in the cystic fibrosis group as a whole [24]. Data coming from recent cohort studies show that liver disease in Cystic Fibrosis patients poses a special threat to their wellbeing and survival. This is not only related to the complications of cirrhosis itself; affected children tend to have higher Shwachman scores and worse pulmonary function suggesting a synergistic effect between liver and lung disease [22, 25]. In fact, improvement in the severity of respiratory disease is well documented after liver transplantation in many of those patients [24, 26]. Altogether, approaching a child suffering from variceal bleeding in the context of Cystic Fibrosis should be tailored to each specific case. Endoscopic treatment should be offered to all, being especially useful in the context of acute hemorrhage. However, concern remains over the long-term endoscopic treatment due to the need for multiple anesthetics procedures, and the possible development of pulmonary complications from portal hypertension itself. In patients with relatively well-preserved liver and lung functions, a selective portocaval shunt (or a TIPS, when feasible) could offer many years of benefit without compromising the outcome [23, 27]. Patients with advanced liver disease, or severe and refractory bleeding, with good pulmonary function are probably best managed with liver transplantation [24, 26, 28]. Results of combined liver-lung transplantation are currently not encouraging; hence waiting for advanced lung disease before deciding to go for liver transplantation does not seem to be advisable [29]. 

## 3. Other Etiologies of Portal Hypertension

### 3.1. Noncirrhotic Portal Hypertension (Hepatoportal Sclerosis)

This presinusoidal type of portal hypertension is produced by intimal thickening of small intrahepatic portal vein radicles. The clinical picture resembles that of prehepatic portal vein obstruction but with a patent (an even, dilated) portal vein on ultrasound. Well-tolerated variceal bleeding and hypersplenism have been reported in this syndrome mainly described in Asian patients [30]. Recent reports coming from western-country children surviving from acute leukemia treated with 6-thioguanin highlights the alleged toxin exposure as one of the possible causes of the endothelial damage [31]. Management of these patients follows the same rules applied for portal vein obstruction.

### 3.2. Postnecrotic Cirrhosis

*Chronic hepatitis associated to HBV or HCV* infection can rarely present in the first two decades of life with a picture of portal hypertension secondary to cirrhosis. Management is not different from that in adult patients. Children exhibit better responses rates to antiviral treatment; thereby there is better control of complications, including those of cirrhosis [32–34].

*Autoimmune hepatitis* is the most common cause of postnecrotic cirrhosis in children. Appropriate treatment with immunosuppressive drugs usually results in control and regression of fibrosis in most patients. A small percentage, however, progresses to decompensated cirrhosis and hemorrhagic complications; these should be managed in a staggered manner according to the medium-term prognosis of the disease, from endoscopic treatment to liver transplantation in end-stage patients [35].

*Alpha-1-antitrypsin deficiency *produces a picture combining findings of cholestatic and postnecrotic cirrhosis. It is the most common indication for liver transplantation from metabolic diseases in the Western hemisphere. Although some improvement of liver function tests has been reported with the use of ursodeoxycholic acid, at the present, there is no effective treatment for this condition, and management of affected patients is restricted to the complications of ongoing cirrhosis, using the same principles described for other etiologies [36].

*Budd-Chiari Syndrome* encompasses a series of different causes producing obstruction to the hepatic venous outflow. These patients tend to present with hepatomegaly and ascitis rather than with variceal hemorrhage, but those developing secondary cirrhosis can experiment bleeding from esophageal varices. Management is very complex, strongly influenced by the clinical picture (acute versus chronic), etiology, and extent of the liver damage. In contrast with portal vein obstruction, most Budd-Chiari patients have an associated thrombophilic state that has to be accurately investigated and treated [37].

## 4. Treatment

### 4.1. Prevention of the First Bleed (Primary Prophylaxis)

Avoiding the morbidity and mortality associated with the first bleed from esophageal varices is the rationale behind primary prophylaxis. Clear recommendations exist for the adult population, but unfortunately this is not the case for pediatric patients [38]. Application of such strategy should comply with two premises: correct identification of the population “at risk” and availability of an effective treatment. In spite of many efforts, achieving the first goal has been elusive, owing to the heterogeneity of the population with portal hypertension in pediatric ages [39]. Stratifying patients at risk according to specific etiologies could be the best way to manage this problem [17]. Regarding the second goal, the absence of controlled randomized trials in primary prophylaxis of esophageal varices bleeding in children makes any recommendation problematic and debatable. Low number of patients and difficulties in recruitment are major obstacles to the realization of such studies, as seen with the use of propranolol in children, which is in strong contrast to the adult population. A group of expert analyzed possibilities on primary prophylaxis of variceal hemorrhage in children, concluding that future research should focus on the natural history, diagnosis of varices, prediction of variceal bleeding, and explore therapeutic efficacy of different protocols [40]. 

Currently, it remains intuitive to offer endoscopic obliteration to patients with high-risk varices who had never bled, preferably by band ligation. Endoscopic examination should be only offered to patients when decision to proceed with sclerotherapy or banding has already been taken in advance [5, 20].

Data in children with cirrhosis secondary to biliary atresia showed that esophageal varices developed very early in life in 70% of them. In addition, endoscopic signs indicating a high risk of mediate bleeding were found in 30% of those with esophageal varices [20]. Another recent study, on a similar population, showed that grade II-III varices developed with similar frequency after failed and successful portoenterostomy, but, following failed portoenterostomy, esophageal varices were encountered significantly earlier [41]. The authors recommended that after failed portoenterostomy surveillance should start early, for example, at six months of age [41].

 There are different approaches in the care of children at risk for esophageal varices bleeding among pediatric gastroenterologists, most of them based on personal preferences and local expertise rather than strong evidence. In addition, attitudes from parents could be different from those of physicians; a high percentage of them would accept an endoscopy to be carried out in their children if a prophylactic treatment can avoid bleeding or even to establish the current risk of bleeding in the absence of treatment [42]. 

### 4.2. Acute Bleeding

Acute bleeding is the most feared complication of portal hypertension, with an associated mortality up to 20%, mainly in patients with affected liver function [43]. As a consequence, focus on treatment has been directed to the control of hemorrhagic episodes, reaching a rate of success higher than 90% in recent years.

Volume resuscitation initiated without delay, should restore hemoglobin levels to around 8 g%, and insure good perfusion of vital organs with plasma expanders. Overzealous use of volume/plasma expanders should be avoided, however, because of the theoretical risk of rebound portal hypertension and rebleeding [38].

Antibiotics directed at the intestinal flora should be part of the treatment from the beginning [38], as well as vasoactive drugs, preferably by the intravenous route. Among many drugs tested in adult patients, octreotide has been the most widely used in children, at a dose of 1-2 ug/Kg by bolus over 20 minutes, followed by continuous infusion at 2 ug/Kg/h, maintained for 2 to 5 days [44]. Its use in this setting has been advocated to promote easier and safer endoscopic procedures [20].

Once stabilized, patients should be treated by direct approach of the varices, either with band ligation or sclerosant injection. Both treatments are highly effective in controlling the acute episode, and the choice of one particular method depends on the local expertise and other technical issues. In a general sense, endoscopic variceal ligation is preferred in most cases, owing to its simplicity and lower rate of complications, but sclerotherapy is probably easier to implement during active bleeding, and is the best option in small children [45, 46]. Ideally, the operator should master both techniques and have all appropriate tools available during the procedure.

Despite the high rate of success achieved with these approaches: in 5 to 10% of cases bleeding cannot be controlled, and rescue therapy is needed, usually after the failure of a second attempt by endoscopy. This rescue therapy involves a surgical option, or a radiological approach (TIPS), when feasible. Once again, both procedures are equally effective, but when used in an emergency scenario their results are less satisfactory [47]. TIPS has the advantage of avoiding a laparotomy, but its availability is limited to specialized services and is not suitable for small children, especially in cases of portal vein obstruction or biliary atresia, which are the main causes of variceal hemorrhage among pediatric patients [38]. The choice of the surgical technique, on the other hand, depends on the medium-term prognosis of the disease. Shunting procedures are preferred in patients with relatively well-preserved liver function, like those with portal vein obstruction, congenital hepatic fibrosis, or compensated cirrhosis. Liver transplantation needs to be considered for children with more advanced disease.

### 4.3. Prevention of Rebleeding (Secondary Prophylaxis)

Once the first bleeding has occurred, there is a substantial risk for rebleeding in the next years; consequently, eradication of esophageal varices becomes a logical goal. Endoscopic variceal ligation and sclerotherapy have been reported to be equally successful in achieving this. Variceal ligation is usually preferred because of its reported simplicity, lesser number of sessions needed, and a safer profile when compared to sclerotherapy [45, 46]. Both techniques are complementary and have been used even in primary prophylaxis with good results [5, 20].

An observational study in children with portal hypertension, of several different etiologies, showed a benefit of secondary prophylaxis in avoiding esophageal varices bleeding. In this study, the use of propranolol did not affect results of endoscopic prophylaxis [48]. In contrast, a large study including mainly adolescents did not find differences between propranolol and endoscopic ligation in the recurrence of bleeding [49].

Longer followup of endoscopic treatments is available, showing recurrence of esophageal varices in 40% of the patients, with a tendency to worsening of gastric varices, portal hypertensive gastropathy, and rising incidence of ectopic varices, all of them representing a more difficult problem to solve [50]. Progression of the spleen size and late incidence of complications like portal cholangiopathy in patients with portal obstruction, formerly considered a rare entity, affect children quality of life. Moreover, for these complications endoscopic treatments are clearly unsuitable [51]. In those cases, or when hemorrhagic episodes are refractory to other treatments, surgery becomes the only option [52]. 

Shunt procedures could be classified as total, partial, and selective. Total portosystemic shunts are those more than 10 mm in diameter, constructed between the main veins of the portal system and the inferior vena cava. They provide excellent control of hemorrhages and ascitis, but at the high cost of encephalopathy, and are rarely used in children. Partial shunts comprises portocaval or mesocaval anastomoses of 8 mm in diameter or less, allowing part of the portal flow to reach the liver sinusoids, and thus reducing the risk of systemic complications without losing efficacy for the prevention of further bleeding. This type of shunts has been widely used in children employing the internal jugular vein as a graft, with excellent results [53, 54]. Selective shunts are constructed by the anastomoses of the splenic vein to the left renal vein, thereby decompressing gastroesophageal varices through the short gastric veins (distal splenorenal shunt), and maintaining portal perfusion to the liver. Spleno-renal shunts achieve good hemorrhagic control and reduce systemic complications. 

Surgical shunts have gained renewed interest in the management of portal hypertension in children with good liver function, in view of better results obtained with the refinement of surgical techniques driven by the development of liver transplantation programs, and the emergence of nonhemorrhagic complications after successful eradication of esophageal varices.

The mesenteric-left portal vein bypass (Rex shunt) is constructed between the superior mesenteric vein and the recessus of Rex at the level of intrahepatic left branch of portal vein. Originally developed to treat patients who have portal vein thrombosis after liver transplantation, it was extended immediately to the treatment of children with extrahepatic portal vein obstruction, allowing them for the first time to reach a real “cure” for their disease. In fact, when successful, it can restore the normal flow to the liver with normalization of hematological tests [55]. Availability of this technique is promoting a change of paradigm in the treatment of portal vein obstruction, towards an early indication of surgery, before progressive fibrosis of the main portal vein branches precludes the feasibility of such anastomoses [56]. The percentage of children with portal obstruction who can benefit for a meso-Rex shunt is still unknown. 

Recent data coming from pediatric series, albeit small in number of patients, have reproduced the rates of success obtained in adult patients, making TIPS a good option even in small children and expanding indications to postransplant portal hypertension, and children with portal vein obstruction with a favorable anatomy [57]. Future studies will clarify the role of this therapy in the management of pediatric portal hypertension [58].

## 5. Summary

Treatment of hemorrhagic complications from portal hypertension in children has its own specificities because of the different etiologies involved, and the natural history of these disorders compared to adults (Table 1). Size constraints can also be anticipated in smaller patients. Despite that, considerable progress has been achieved in the last years, mainly derived from better control of bleeding from esophageal varices. Longer followup, however, uncovers new complications for which endoscopic treatment is inappropriate, promoting a renewed interest on surgical approaches. As a general principle, management of portal hypertension in children rests on two main characteristics: the etiology of the portal hypertension and the age of the patient. 

## Figures and Tables

**Table 1:** Common causes of portal hypertension in children and suggested management.

Cause	Treatment	Comment
	(1) Endoscopic	
Portal vein obstruction	(2) Meso-Rex shunt	Endoscopic treatment consists on elastic banding or sclerotherapy
	(3) DSR or mesocaval shunt	

Biliary atresia	(1) Endoscopic	Screening at age of 1, prophylaxis in high-risk varices
	(2) Liver transplantation	

	(1) Endoscopic	Need repetitive anesthetics
Cystic fibrosis	(2) DSR or meso-caval shunt	Risk of pulmonary complications and worsening encephalopathy
	(3) Liver transplantation	When good respiratory function

	(1) Endoscopic	
Congenital hepatic fibrosis	(2) DSR or meso-caval shunt	When recurrent cholangitis (need to consider liver and kidney tranplantation)
	(3) Liver transplantation	

	(1) Endoscopic	
Other cirrhosis	(2) DSR or meso-caval shunt	If good liver function
	(3) Liver transplantation	In end-stage liver disease

DSR: distal spleno-renal shunt.
